# Enzyme characterization of lactic acid bacteria isolated from duck excreta

**DOI:** 10.14202/vetworld.2024.143-149

**Published:** 2024-01-20

**Authors:** Dini Dwi Ludfiani, Widya Asmara, Forita Dyah Arianti

**Affiliations:** 1 Research Center for Sustainable Production Systems and Life Cycle Assessment, National Research and Innovation Agency (BRIN), Tangerang Selatan, Indonesia; 2 Department of Microbiology, Faculty of Veterinary Medicine, Universitas Gadjah Mada, Yogyakarta, Indonesia

**Keywords:** API ZYM, duck, enzyme, excreta, lactic acid bacteria

## Abstract

**Background and Aim:**

The production of lignocellulosic biomass waste in the agricultural sector of Indonesia is quite high annually. Utilization of lignocellulosic biomass waste through fermentation technology can be used as feed and biofuel. Fermentation technology requires the involvement of micro-organisms such as bacteria (lactic acid bacteria or LAB). LABs can be isolated from various sources, such as duck excreta. However, there have not been many reports of LAB from duck excreta. The present study aimed to characterize LAB enzymes isolated from duck excreta and obtain LAB enzymes with superior fermentation properties.

**Materials and Methods:**

A total of 11 LAB cultures obtained from duck excreta in Yogyakarta, Indonesia, were tested. Enzyme characterization of each LAB was performed using the API ZYM kit (BioMérieux, Marcy-I’Etoile, France). The bacterial cell suspension was dropped onto the API ZYM™ cupule using a pipette and incubated for 4 h at 37°C. After incubation, ZYM A and ZYM B were dripped onto the API ZYM cupule, and color changes were observed for approximately 10 s under a strong light source.

**Results:**

Esterase activity was moderate for all LABs. The activity of α-chymotrypsin, β-glucuronidase, α-fucosidase, and α-mannosidase was not observed in a total of 10 LAB. The phosphohydrolase and amino peptidase enzyme activity of seven LABs was strong. Only six LAB samples showed protease activity. The glycosyl hydrolase (GH) activity was observed in a total of 8 LAB, while the activity of 2 LAB was strong (*Lactococcus lactis* subsp. *lactis* K5 and *Lactobacillus brevis* M4A).

**Conclusion:**

A total of 2 LABs have superior properties. *L. lactis* subsp. *lactis* K5 and *L. brevis* M4A have a high potential to be used in fermentation. They have the potential for further research, such as their effectiveness in fermentation, lignocellulose hydrolysis, feed additives, molecular characterization to detect specific enzymes, and their specific activities.

## Introduction

The annual production of lignocellulosic biomass waste in Indonesia’s agricultural sector is quite high, but its utilization is not optimal. Only 10%–20% of lignocellulosic biomass waste is burned or dumped [1, 2]. Processing lignocellulosic biomass waste into several value-added products is a good solution for agriculture, livestock, industry, and the environment. Lignocellulosic biomass waste can be used as feed, fertilizer, and biofuel by fermentation technology. Fermentation technology requires micro-organisms, such as lactic acid bacteria (LAB), yeast, or fungi, to degrade complex compounds (polysaccharides) into simple compounds (monosaccharides) [3]. LAB is a heterogeneous group of bacteria with significant fermentation capacities [4] and is generally recognized as safe to use [5]. Gram-positive, microaerophilic, non-spore-forming, cocci or rod-shaped LABs are the characteristics. LABs consist of homofermentative LAB (homo-LAB) and heterofermentative LAB (hetero-LAB) [6]. Homo-LAB produces lactic acid as the main metabolic product, whereas hetero-LAB produces lactic acid and carbon dioxide (CO_2_), as well as acetic acid and ethanol [7]. Homo-LABs are widely used in industry due to their high lactic acid production. Hetero-LABs are used for biofuel production because they produce acetic acid and butanol/ethanol. LABs are a potential sweetener, producing ethanol, synthesis of biodegradable plastic polymers, and exopolysaccharides [8]. LAB can be used as a bio-preservative, enzyme, medicine, bio-additive [9], and green biorefineries [10] and have many beneficial properties.

LAB can be found and isolated from feces [11–13] and excreta [14–16] from various sources [17], and different environments [18]. Micro-organisms present in the feces reflect groups of micro-organisms present in the gastrointestinal tract (GIT) [19]. The previous studies have reported that LAB can be obtained from the GIT of duck. LABs were isolated from the small intestine [20, 21], cecum, and colon of Muscovy ducks [22] or from the crop until the cecum of Aceh ducks [23]. Ducks can survive in various agroclimatic conditions [24]. Therefore, ducks have the ability to digest fiber, and the caecum of ducks can absorb 20% of fiber [25]. Fiber increases the weight and length of the GIT [26] and modulates the gut microbiome [27]. The cecum of ducks is highly developed and plays a significant role in microbial digestion. Cecum micro-organisms play an important role in the physiological processes of growth, metabolism [28], and host health maintenance [29]. Micro-organisms of the caecum have superior properties in fiber degradation, and the isolation of bacteria from duck excreta is expected to produce bacteria with superior properties similar to those found in the caecum. It can be used in lignocellulosic biomass bioprocessing technology (particularly fermentation).

Isolation and characterization of LAB from duck excreta have not been widely reported, and the novelty of this study was to characterize LAB from duck excreta based on enzyme profiles. Many previous studies have reported on the isolation and characterization of LAB from human feces [30, 31], animal feces [32–34], and chicken excreta using general probiotic criteria. Ludfiani *et al*. [35] tested LAB isolated from chicken and duck excreta for sensitivity to antibiotics. In this study, enzyme characterization of LAB was performed to obtain LAB with superior hydrolase activity that can be used in lignocellulosic biomass fermentation applications. Enzyme characterization was previously carried out on fecal samples from dogs suffering from acute non-hemorrhagic diarrhea treated with *Enterococcus faecium* DSM 32820 to determine the effectiveness of the treatment and changes in the microbial community [36]. We screened LAB from raw milk and dairy products to obtain LAB with high α-galactosidases production [37]. LAB obtained from artisanal cheese from Caucasus has the potential to produce bioamines (tyramine and putrescine) [38]. LAB from various dairy sources was screened for β-galactosidase activity for whey acid valorization [39]. According to Vieco-Saiz *et al*. [40], LAB exhibits specific enzymatic properties. Hydrolytic enzymes (hydrolases) are enzymes that catalyze the cleavage of substrates, consisting of glycoside hydrolases (glycosidases), esterases, lipases, nucleotidases, peptidases, and phosphatases. The potential hydrolase activity of LAB isolated from duck excreta has not been reported. Bacteria and actinomycetes isolated from the stump and soil of wilted banana plants can produce hydrolases [41]. Extremophile bacteria isolated from a hot spring in South Sulawesi demonstrated hydrolase activity [42]. Yeast isolated from the GIT of *Gymnopleurus sturmi* and ruminant feces showed cellulase production [43].

This study aimed to characterize LAB enzymes isolated from duck excreta and obtain LAB with superior fermentation properties. Bacteria with superior properties are expected to optimize the processing of lignocellulose biomass waste as feed, food, and biofuel (bioethanol) through fermentation technology.

## Materials and Methods

### Ethical approval

This study was approved by Research Ethics Committee, Faculty of Veterinary Medicine, Universitas Gadjah Mada, Indonesia (Ethical clearance number: 0028/EC-FKH/Int./2020).

### Study period and location

This study was conducted in the Microbiology Laboratory of the Center for Food and Nutrition Studies, Universitas Gadjah Mada, Indonesia. LAB cultures were obtained from duck excreta at duck farms in Bantul, Yogyakarta.

### Bacteria

LAB cultures were obtained from Dini Dwi Ludfiani and Widya Asmara, the bacteria isolated from duck excreta in Yogyakarta, Indonesia. A total of 11 LABs were tested, of which nine have been identified biochemically and molecularly (*Lactobacillus brevis* M4A, *Lactococcus raffinolactis* H12, *Lactobacillus brevis* H23*, Lactobacillus brevis* H54, *Lactobacillus pentosus* 3B, *Lactococcus lactis* subsp. *lactis* K5, *Lactobacillus plantarum* BJ3, *Lactococcus lactis* BJ11, and *Lactobacillus plantarum* K3.0).

### Enzymatic profile characterization

Enzyme characterization of each LAB was performed using the API ZYM kit according to the manufacturer’s protocol (BioMérieux, Marcy-I’Etoile, France). Bacterial cell suspension from culture media on deMan, Rogosa, Sharpe (MRS) agar (Merck, Darmstadt, Germany) overnight (turbidity McFarland tube No. 5) was dropped onto the API ZYM cupule using a pipette (65 μL), then the plastic lid was placed on the tray and incubated for 4 h at 37°C. The tray was not illuminated in bright light. ZYM A (tension active agent) and ZYM B (diazonium salt) were then dripped onto the API ZYM cupule, and the tray was placed under a strong source of light for about 10 s. Color changes were observed and compared with the API ZYM color scale reference from 0 (no activity) to 5 (maximum activity) [44]. The enzyme profiles are shown in Table-1. All LAB enzyme profile descriptions were based on the color change in the cupule.

**Table-1:** Enzyme profile of API ZYM kit.

S. No.	Enzyme assayed for	pH	Substrates
1.	Control		
2.	Alkaline phosphatase	8.5	2-naphthyl phosphate
3.	Esterase (C4)	6.5	2-naphthyl butyrate
4.	Esterase lipase (C8)	7.5	2-naphthyl caprylate
5.	Lipase (C14)	7.5	2-naphthyl myristate
6.	Leucine arylamidase	7.5	L-leucyl-2-naphthylamide
7.	Valine arylamidase	7.5	L-valyl-2-naphthylamide
8.	Cystine arylamidase	7.5	L-cystyl-2-naphthylamide
9.	Trypsin	8.5	N-benzoyl-DL-arginine-2-naphthylamide
10.	α-chymotrypsin	7.5	N-glutaryl-phenylalanine-2-naphthylamide
11.	Acid phosphatase	5.4	2-naphthyl phosphate
12.	Naphthol-AS-BI-phosphohydrolase	5.4	Naphthol-AS-BI-phosphate
13.	α-galactosidase	5.4	6-Br-2-naphthyl-αD-galactopyranoside
14.	β-galactosidase	5.4	2-naphthyl-αD- galactopyranoside
15.	β-glucuronidase	5.4	Naphthol-AS-BI-αD-glucuronide
16.	α-glucosidase	5.4	2-naphthyl-αD-glucopyranoside
17.	β-glucosidase	5.4	6-Br-2-naphthyl-βD-glucopyranoside
18.	N-acetyl-α-glucosaminidase	5.4	1-naphthyl-N-acetyl-βD-glucosaminide
19.	α-mannosidase	5.4	6-Br-2-naphthyl-αD-mannopyranoside
20.	α-fucosidase	5.4	2-naphthyl-αL-fucopyranoside

Source: BioMérieux, Marcy-I’Etoile, France

## Results

The enzyme activity of LAB varied (Table-2). The esterase activity of all LAB was moderate. All LAB samples did not show α-chymotrypsin, β-glucuronidase, α-fucosidase, and α-mannosidase activity (except LAB BJ7.0). The phosphatase activity (Naphthol-AS-BI-phosphohydrolase) and amino peptidase activity (leucine arylamidase and valine arylamidase) of LAB were strong, except that of *L. plantarum* K3.0, *L. raffinolactis* H12, LAB BJ1, and LAB BJ7 showed moderate phosphohydrolase activity and low amino peptidase activity. Only six LAB showed protease activity, but they had low activity. The glycosyl hydrolase activity of LAB varied. Glycosyl hydrolase activity of *L. lactis* subsp. *lactis* K5 and *L. brevis* M4A was strong, whereas that of *L. plantarum* BJ3, *L. lactis* BJ11, and LAB BJ1 was moderate.

**Table-2:** Enzyme profile of LAB.

Enzyme profile	M4A	H23	H54	3B	BJ3	K3.0	H12	K5	BJ11	BJ1.0	BJ7.0
Phosphatase											
Alkaline phosphatase	−	−	+	+	+	+	+	+	+	+	+
Acid phosphatase	+	+	+	+	+	+	+	+	+	+	+
Naphtol-AS-BI-phosphohydrolase	+	+	+	+	+	+	+	+	+	+	+
Esterase											
Esterase (C4)	−	−	+	+	+	+	+	+	+	+	+
Esterase lipase (C8)	+	+	+	+	+	+	+	+	+	+	+
Lipase (C14)	+	+	+	+	+	−	+	+	+	+	+
Amino peptidase											
Leucine arylamidase	+	+	+	+	+	+	+	+	+	+	+
Valine arylamidase	+	+	+	+	+	−	+	+	+	+	+
Cystine arylamidase	+	+	+	+	+	−	−	+	+	+	+
Protease											
Trypsin	+	+	+	+	+	−	−	+	−	−	−
α-chymotrypsin	−	−	−	−	−	−	−	−	−	−	−
Glycosyl hydrolase											
α-galactosidase	+	−	−	−	−	−	−	−	−	−	−
β-galactosidase	−	+	+	+	+	−	−	+	+	+	−
β-glucuronidase	−	−	−	−	−	−	−	−	−	−	−
α-glucosidase	−	+	−	−	+	−	−	+	+	+	−
β-glucosidase	+	+	+	+	+	−	−	+	+	+	−
N-acetyl-β-glucosaminidase	+	−	−	−	+	−	−	+	+	+	−
α-mannosidase	−	−	−	−	−	−	−	−	−	−	+
α-fucosidase	−	−	−	−	−	−	−	−	−	−	−

LAB=Lactic acid bacteria

## Discussion

Hydrolytic enzyme plays a central role in biochemistry (catalysis) [45]. Bacteria produce a number of enzymes that play specific roles. The use of the API ZYM in this study helps to quickly characterize bacterial strains with beneficial potential. According to Muñoz-Quezada *et al*. [46], the API ZYM system enables strains to be characterized according to their enzymatic type and level of activity. API ZYM is a semi-quantitative micromethod used in systematic and rapid studies to evaluate enzyme profile [47]. API ZYM is a class of hydrolases consisting of phosphatases, esterases, amino peptidases, proteases, and glycosyl hydrolases. Hydrolases play a role in the cleavage of molecular bonds, such as ester, glycosidic, ether, peptide, and phosphatase bonds [48]. Enzymatic hydrolysis is required in the degradation of complex compounds such as lignocellulose. Hydrolytic enzymes are used for food, feed, biofuel, paper, and pulp in up to 75% of all industries [49].

In this study, the seven LABs showed strong catabolic activities (production of amino peptidases and phosphohydrolases) and low esterase lipase activity. Similar to Rondón *et al*. [50], the activity of amino peptidase, phosphatase, and phosphohydrolase from *Lactobacillus salivarius* is strong. All *Lactobacillus* spp. strains studied by Pisano *et al*. [51] showed high amino peptidase activity (leucine and valine arylamidase). LABs with high peptidase and low esterase/lipase activity may be useful in fermented dairy milk (cheese production) for improving the texture and reducing bitterness [52]. Amino peptidases are exopeptidases that cleave amino acid residues from the N-terminus [53]. This enzyme is widely used in the food industry for the hydrolysis of organophosphate compounds and biopeptide and amino acid synthesis [54].

Proteolytic activity plays an important role in fermentation through secondary catabolic reactions, especially for organoleptic properties, flavor development and texture improvement [55, 56]. Proteolytic activities of microbes produce different hydrolysis products, and lipases produce esters, aldehydes, ketones, lactones, and alcohols. These factors contribute to different sensory characteristics [57]. The proteolytic system of LAB consists of cell envelope proteinase, short peptide and amino acid transport systems, and a multitude of intracellular peptidases [58].

LAB can efficiently degrade polysaccharides using carbohydrate active enzyme (CAZyme). CAZyme acts synergistically to breakdown cell wall components (such as cellulose and hemicellulose) [59]. CAZyme plays a role in the biosynthesis, modification, binding, and catabolism of carbohydrates, which are divided on the basis of their catalytic activities [60]. CAZyme plays an important role in the synthesis and degradation of polysaccharides and their derivatives [61]. Based on the CAZymes database (http://www.cazy.org), CAZymes are divided into five categories: (1) glycoside hydrolases (GH), (2) glycosyl transferases (GT), (3) polysaccharide lyases (PL), (4) carbohydrate esterases (CE), and (5) auxiliary activities (AA). GH plays a role in the hydrolysis and/or rearrangement of glycosidic bonds, GT plays a role in the formation of glycosidic bonds, PL plays a role in breaking non-hydrolytic glycosidic bonds, CE plays a role in the hydrolysis of carbohydrate esters, and AA is a redox enzyme that works together with CAZymes. According to Madhavan *et al*. [62], carbohydrase enzymes have a fairly high potential for development because they are widely used in the food, feed, and pharmaceutical industries and are predicted to continue to increase their use in the industry. CAZyme in ruminants degrades lignocellulosic into short-chain fatty acids (acetic acid, propionic acid, and butyric acid) through different metabolic pathways. Acetic acid, butyric acid, and propionic acid are produced through the wood–Ljungdahl pathway, the acetate CoA-transferase pathway, the butyrate kinase pathway, the acrylate pathway, the succinate pathway, and the propanediol pathway [63].

Enzymes that play a role in the degradation of lignocellulosic are lignases or ligninolytic enzymes (such as laccase, manganese peroxidase, lignin peroxidase, and versatile peroxidase), cellulases or cellulolytic enzymes (such as β-glucosidase, endoglucanase, and exoglucanase), and hemicellulases or hemicellulolytic enzymes (such as xylanase, β-glucosidase, acetyl esterase, α-galactosidase, endoglucanase, and mannanase) [64]. Based on the enzyme information in Braunschweig enzyme database, strain *Lactobacillus* spp. and *Lactococcus* spp. produce lignases, cellulases, and hemicellulases (such as lignin peroxidase, cellulase, α-glucosidase, β-glucosidase, β-galactosidase on *L. brevis*; cellulase, β-glucosidase, β-galactosidase on *L. pentosus*; laccase, cellulase, α-glucosidase, β-glucosidase, α-galactosidase, β-galactosidase, α-mannosidase, and N-acetyl-β-glucosaminidase on *L. plantarum*).

Some LAB in this study showed activity of β-galactosidase, α-glucosidase, β-glucosidase, and N-acetyl-β-glucosaminidase, and only *L*. *brevis* M4A showed α-galactosidase activity. The activity of α-galactosidase, β-galactosidase, α-glucosidase, β-glucosidase, and N-acetyl-β-glucosaminidase was similar to that of LAB isolated from silage, milk, and rumen in the study by Colombo *et al*. [52]. Rada [65] reported that some of the LABs were both α-galactosidase and α-glucosidase positive. Some of *Lactobacillus* spp. in study Pisano *et al*. [51] showed activity of α-galactosidase, β-galactosidase, α-glucosidase, β-glucosidase, and N-acetyl-β-glucosaminidase. *L. plantarum*, which was found in fermented food, also showed the highest hydrolase activity, such as β-glucosidase, and it has potency as a biotransformation agent for cellulosic biomass [66].

α-galactosidase plays an important role in the hydrolysis of glycolipids and glycoproteins [67]. Moreover, *Lactobacillus* strains have α-galactosidase that hydrolyzes non-digestible carbohydrates into digestible carbohydrates during fermentation [68]. The β-galactosidase enzyme is generally extracted from *Lactobacillus* [69] and *Pediococcus* probiotic strains and is commonly used in food technology for the hydrolysis of lactose [70]. The β-galactosidase or lactase plays an important role in the hydrolysis of the β-1,4 glycosidic bond between galactose and glucose [71]. β-galactosidase is one of the characteristics of probiotics for increasing lactose tolerance [72]. β-glucosidase acts to cleave cellobiose into glucose, and β-glucosidase belongs to cellulose, which is used for the hydrolysis of biomass [73]. β-glucosidase releases glucose from cellobiose glycosidic bonds in the final step of cellulolysis or during the hydrolysis of lignocellulosic biomass [74–76]. α-glucosidase hydrolyzes the terminal non-reducing end of the α-1,4 linkages of starch and maltose with glucose release [77].

Four LAB in this study were homo-LAB (*L. brevis* H23, *L. brevis* H54, *L. lactis* BJ11, and LAB BJ7.0) and seven LAB were hetero-LAB (*L. brevis* M4A, *L. raffinolactis* H12, *L. pentosus* 3B, *L. lactis* subsp. *lactis* K5, *L. plantarum* BJ3, *L. plantarum* K3.0, and LAB BJ1.0). Homo-LAB produces lactate acid through the Embden–Meyerhof–Parnas and pentose phosphate/glycolic pathways, whereas hetero-LAB produces lactate acid, CO_2_, acetic acid, and ethanol through the phosphoketolase pathway [78].

## Conclusion

A total of 2 LABs had superior properties. *L. lactis* subsp. *lactis* K5 and *L. brevis* M4A have a high potential to be used in fermentation. They have the potential for further research, such as their effectiveness in fermentation, lignocellulose hydrolysis, feed additives, molecular characterization to detect specific enzymes, and their specific activities.

## Authors’ Contributions

DDL: Formal analysis and methodology. WA and FDA: Conception of the study. DDL, WA, and FDA: Drafted, reviewed, and revised the manuscript. All authors have read, reviewed, and approved the final manuscript.
